# Gene Targeting Facilitated by Engineered Sequence-Specific Nucleases: Potential Applications for Crop Improvement

**DOI:** 10.1093/pcp/pcab034

**Published:** 2021-02-26

**Authors:** Daisuke Miki, Rui Wang, Jing Li, Dali Kong, Lei Zhang, Jian-Kang Zhu

**Affiliations:** Shanghai Center for Plant Stress Biology and Center for Excellence in Molecular Plant Sciences, Chinese Academy of Sciences, Shanghai 200032, China; Shanghai Center for Plant Stress Biology and Center for Excellence in Molecular Plant Sciences, Chinese Academy of Sciences, Shanghai 200032, China; University of Chinese Academy of Sciences, Beijing 100049, China; Shanghai Center for Plant Stress Biology and Center for Excellence in Molecular Plant Sciences, Chinese Academy of Sciences, Shanghai 200032, China; University of Chinese Academy of Sciences, Beijing 100049, China; Shanghai Center for Plant Stress Biology and Center for Excellence in Molecular Plant Sciences, Chinese Academy of Sciences, Shanghai 200032, China; University of Chinese Academy of Sciences, Beijing 100049, China; Shanghai Center for Plant Stress Biology and Center for Excellence in Molecular Plant Sciences, Chinese Academy of Sciences, Shanghai 200032, China; University of Chinese Academy of Sciences, Beijing 100049, China; Shanghai Center for Plant Stress Biology and Center for Excellence in Molecular Plant Sciences, Chinese Academy of Sciences, Shanghai 200032, China; Department of Horticulture and Landscape Architecture, Purdue University, West Lafayette, IN 47907, USA

**Keywords:** Crop, Gene targeting, Sequence-specific nuclease

## Abstract

Humans are currently facing the problem of how to ensure that there is enough food to feed all of the world’s population. Ensuring that the food supply is sufficient will likely require the modification of crop genomes to improve their agronomic traits. The development of engineered sequence-specific nucleases (SSNs) paved the way for targeted gene editing in organisms, including plants. SSNs generate a double-strand break (DSB) at the target DNA site in a sequence-specific manner. These DSBs are predominantly repaired via error-prone non-homologous end joining and are only rarely repaired via error-free homology-directed repair if an appropriate donor template is provided. Gene targeting (GT), i.e. the integration or replacement of a particular sequence, can be achieved with combinations of SSNs and repair donor templates. Although its efficiency is extremely low, GT has been achieved in some higher plants. Here, we provide an overview of SSN-facilitated GT in higher plants and discuss the potential of GT as a powerful tool for generating crop plants with desirable features.

## Introduction

The United Nations Food and Agriculture Organization (FAO) estimates that about 10.7% of people in the world are currently suffering from chronic undernourishment. The world population is predicted to rise to 10.5 billion by 2050, at which time we will require 100% more food than is produced today; most of this increase in food production must come from innovative technologies (Hickey et al. 2019). The agricultural industry must radically change if it is to support such a rise in population. Approximately 40% of the agricultural fields in the world are seriously impacted by biotic and abiotic stresses, which cause annual losses of 30–50% of major crops (Pennisi 2010, Kim et al. 2019). Extreme climate-related disasters are also increasing and becoming a leading cause of food shortages. It follows that we must increase the food production capacity of the agricultural sector. One approach is to use genetic engineering to create genetically modified organisms (GMOs) for food production that would be less vulnerable to stress and thus less susceptible to stress-related losses. However, many consumers have concerns about GMO products, mainly because such products harbor transgenes. The ability to manipulate genomic sequences without inserting transgenes would have a large positive impact on plant biotechnology, the agricultural industry and consumers.

**Fig. 1:**
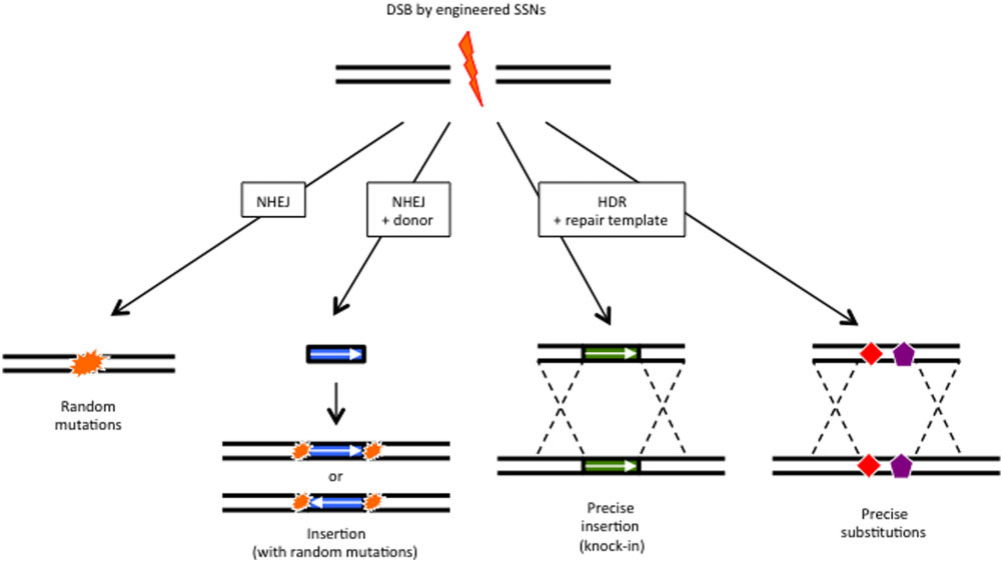
Various DSB repair pathways in higher plants. The primary repair mechanism for site-specific DSBs induced by SSNs is the error-prone NHEJ, which leads to random mutations. The error-free HDR creates precise knock-in or sequence substitutions when a specific repair template is provided.

Although the generation of crop plants with desirable features via precise genome modification is the ultimate goal, precise genome modification remains a difficult task, especially in higher plants. Recently developed genome-editing technologies use engineered sequence-specific nucleases (SSNs), such as zinc finger nuclease (ZFN), transcription activator-like effector nuclease (TALEN) and clustered regularly interspaced short palindromic repeats (CRISPR)/CRISPR-associated protein (Cas) systems, to generate site-specific double-strand breaks ( DSBs) in a variety of organisms. In eukaryotic cells, these DSBs are predominantly repaired via error-prone non-homologous end-joining (NHEJ). The NHEJ pathway generally incorporates short in-del mutations at the target site, although specific knock-ins can be achieved if a donor fragment without homology is provided (Fig. 1). Another repair pathway for DSBs is error-free homology-directed repair (HDR), which creates precise sequence changes (such as knock-ins and substitutions) when a homologous DNA substrate is provided (Fig. 1). HDR occurs primarily during the S and G2 phases of the cell cycle when DNA replication is completed and sister chromosomes are available for repair, while NHEJ is active throughout the cell cycle.

Gene targeting (GT) refers to the precise replacement or knocking-in of a specific sequence at an endogenous locus using homologous recombination (HR). GT was originally established in mammalian cells (Smithies et al. 1985) and is now widely used in the research of many organisms, including *Drosophila* and mammals (Hinnen et al. 1978, Thomas and Capecchi 1987, Chandrasegaran and Carroll 2016). In spite of its importance, GT remains difficult in higher plants because of the plants’ extremely low HR frequency (Fauser et al. 2012). The first GT in higher plants was demonstrated for a kanamycin-resistant gene in tobacco with a frequency of only 10^−^^3^ to 10^−^^6^ (Paszkowski et al. 1988). A complicated, yet more efficient method using positive-negative selection was later developed in rice (Terada et al. 2002) but has only been used to modify a few genes in rice (Shimatani et al. 2014); attempts to apply the method to Arabidopsis (Gallego et al. 1999, Wang et al. 2001) and tobacco (Morton and Hooykaas 1995) were unsuccessful. SSNs can increase the efficiency of GT (Choulika et al. 1995, Chandrasegaran and Carroll 2016, Paquet et al. 2016), and CRISPR/Cas9-assisted HDR has been used to perform GT in various model systems, including human stem cells (Paquet et al. 2016). The introduction of DSBs also increases the frequency of HDR in plants (Puchta et al. 1996, Miki et al. 2017), and recent studies have used SSNs for HDR-mediated GT in Arabidopsis (de Pater et al. 2013, Qi et al. 2013, Baltes et al. 2014, Schiml et al. 2014, Sauer et al. 2016, Zhao et al. 2016, de Pater et al. 2018, Hahn et al. 2018, Miki et al. 2018, Wolter et al. 2018, Wolter and Puchta 2019, Merker et al. 2020a, Peng et al. 2020), tobacco (Wright et al. 2005, Cai et al. 2009, Townsend et al. 2009, Li et al. 2013, Zhang et al. 2013, Baltes et al. 2014, Schneider et al. 2016, Hirohata et al. 2019, Huang et al. 2021), soybean (Li et al. 2015), tomato (Čermák et al. 2015, Dahan-Meir et al. 2018, Danilo et al. 2019, Vu et al. 2020), rice (Shan et al. 2013, Shan et al. 2014, Endo et al. 2016, Li et al. 2016, Sun et al. 2016, Begemann et al. 2017, Butt et al. 2017, Wang et al. 2017, Li et al. 2018a, Li et al. 2018b, Li et al. 2019, Li et al. 2020a, Li et al. 2020b, Lu et al. 2020; Wang et al. 2017), maize (Shukla et al. 2009, Ainley et al. 2013, Svitashev et al. 2015, Svitashev et al. 2016, Shi et al. 2017, Barone et al. 2020), wheat (Gil-Humanes et al. 2017), potato (Butler et al. 2016), barley (Budhagatapalli et al. 2015), flax (Sauer et al. 2016) and cassava (Hummel et al. 2018) (Table 1). The molecular machinery and widespread application of SSNs have been described in many primary publications and reviews (Gaj et al. 2013, Mao et al. 2019, Li and Xia 2020b, Zhu et al. 2020, Capdeville et al. 2021). This review focuses on recent progress in the use of SSNs to achieve GT in higher plants.

**Table 1:** SSN-mediated gene-targeting in higher plants

Plant species	SSN	Promoter	Target gene	Transformation	Virus replicon	Donor excision	Efficiency (%)	Screening	References
Arabidopsis	ZFN	CaMV 35S	PPO substitution	Agrobacterium			0.27	Butafenacil resistance	de Pater et al. (2013)
	ZFN	Estradiol inducible	ADH1 68 bp KI	Protoplast electroporation			0.11–5.32	PCR	Qi et al. (2013)
	ZFN	CaMV 35S	35S::GFP Replace to Hpt by NHEJ	Agrobacterium		By ZFN	4.8	Hygromycin resistance	Weinthal et al. (2013)
	ZFN	Estrogen inducible	ADH1 18 bp KI	Bombardment GT in leaf somatic tissue	CaLCuV		4.3	PCR	Baltes et al. (2014)
	SpCas9	PcUbi4-2	ADH1 NptII-KI	Agrobacterium		By SpCas9	0.14 (2/1,400)	PCR Allyl alcohol	Schiml et al. (2014)
	SpCas9	CaMV 35S	AtTFL Replace to GFP	Agrobacterium		By SpCas9	0.8 (4/500)	PCR	Zhao et al. (2016)
	TALEN	Mannopine synthase	BFP transgene substitution	Protoplast electroporation ssODNs donor			0.3–1	Flow cytometry	Sauer et al. (2016)
	SpCas9	Mannopine synthase					∼6		
	SpCas9	PcUbi4-2	PPO substitution	Agrobacterium	Gemini virus		0.016 (1/6,000)	Butafenacil resistance	de Pater et al. (2018)
	SaCas9	PcUbi4-2 YAO EC1.1 promoter with EC1.2 enhancer	ALS substitution	Agrobacterium		By SaCas9	0.14–0.3 0.08 0.07–0.97	Imazapyr resistance	Wolter et al. (2018)
	SpCas9	PcUbi4-2	gl1 mutant 10 bp insertion	Agrobacterium		By SpCas9	T2 chimeric T3 full GT	Trichome phenotype	Hahn et al. (2018)
	SpCas9	DD45	ROS1 DME KI and substitution	Agrobacterium			5.3–9.1	PCR	Miki et al. (2018)
	SaCas9	EC1.1 promoter with EC1.2 enhancer	ALS substitution	Agrobacterium		By SaCas9	0.95–0.98	Imazapyr resistance	Wolter and Puchta (2019)
	LbCas12a					By LbCas12a	1.48		
	SpCas9	DD45 promoter with omega enhancer	ROS1 GFP-KI	Agrobacterium			2.4	PCR	(Peng et al. 2020)
	ttLbCas12a	EC1.1 promoter with EC1.2 enhancer	ALS substitution	Agrobacterium		By ttCas12a	1.34	Imazapyr resistance	Merker et al. (2020b)
Tobacco	ZFN	An artificial high expression promoter	Truncated gus and nptII transgenes	Protoplast electroporation			0.078	Kanamycin resistance GUS expression	Wright et al. (2005)
	ZFN	CaMV 35S	ALS substitution	Protoplast electroporation			0.2–4	Herbicide resistance	Townsend et al. (2009)
	ZFN	CaMV 35S	CEN50 35S::PAT-KI	Agrobacterium			5.2–10.8	Bialaphos resistant	Cai et al. (2009)
	SpCas9	CaMV 35S	PDS AvrII (6 bp)-KI	Protoplast electroporation (no plant regeneration)			9–10.7	RFLP	Li et al. (2013)
	TALEN	CaMV 35S	ALS YFP-KI	Protoplast electroporation			14	Flow cytometry	Zhang et al. (2013)
	ZFN	CaMV 35S	Truncated 35s-gus-nptII transgene	Agrobacterium infiltration	BeYDV			GUS staining	Baltes et al. (2014)
	SpCas9	G10-90	ALS (SuRB) substitution and 35S::HPT KI	Agrobacterium			0.046–0.29	Chlorsulfuron and hygromycin resistance	Hirohata et al. (2019)
	SaCas9	PcUbi4-2	ALS (SuRB) substitution	Agrobacterium		By SaCas9	6–13	Imazapyr resistance	Huang et al. (2021)
	ttLbCas12a					By ttLbCas12a	8–23		
(BY-2 cell)	ZFN	CsVMV (Cassava vein mosaic virus promoter)	AHAS Replace to NptII	Bombardment			1.2 (16/1,326)	Kanamycin resistance	Schneider et al. (2016)
	ZFN	No description	Truncated nptII and DsRed transgene 20K bp-KI	Bombardment			5.5 (18/327) 3 (1/33)	Kanamycin resistance DsRed fluorescence	(Schiermeyer et al., 2019)
Soybean	SpCas9	Soybean Elongation factor1 alpha2 (EF1A2) promoter	DD20 DD43	Bombardment			3.8–4.6	PCR	Li et al. (2015)
Tomato	TALEN	CaMV 35S	ANT1 NptII-KI	Agrobacterium	BeYDV		9.56	Kanamycin resistance	Čermák et al. 2015
	SpCas9	CaMV 35S					3.65–11.66		
	SpCas9	SlUbi10	CRTISO	Agrobacterium	BeYDV		25	Red fruit phenotype	Dahan-Meir et al. (2018)
	SpCas9	Ubi4	ALS1	Agrobacterium			12.7 (31/244) 38 (12/31) T-DNA free	Chlorsulfuron resistance	Danilo et al. (2019)
	LbCas12a	CaMV 35S with AtUbq10 5′ UTR	SlHKT1;2 substitution	Agrobacterium	BeYDV		0.66 (1/150)	PCR	Vu et al. (2020)
Rice	SpCas9	CaMV 35S	OsPDS	Protoplast electroporation (no plant regeneration)			6.9 (2/29)	RFLP	Shan et al. (2013)
	SpCas9	CaMV 35S	OsMPK2	Protoplast electroporation (no plant regeneration)			∼1	RFLP	Shan et al. (2014)
	SpCas9	CaMV 35S	OsALS substitution	Agrobacterium Sequential transformation lig4 mutant			0.147–1	BS resistance	Endo et al. (2016)
	SpCas9	Maize Ubi	OsALS substitution	Agrobacterium			33.4 (80/240)	BS resistance	Sun et al. (2016)
	TALEN	CaMV 35S and Maize Ubi1	OsALS substitution	Bombardment Regenerate plants			1.4–6.3	BS resistance	Li et al. (2016a) and Li et al. (2016b)
	SpCas9	Maize Ubi	OsEPSPS NHEJ-mediated KI	Bombardment			2–2.2	PCR	Li et al. (2016a) and Li et al. (2016b)
	LbCas12a	CaMV 35S	OsCAO1	Bombardment Regenerate plants			0–3	PCR	Begemann et al. (2017)
	FnCas12a						3–8		
	SpCas9	CaMV 35S	OsAct1 GST GFP- and NptII-KI	Agrobacterium Sequential transformation	WDV		4.7–19.4	Kanamycine resitance	Wang et al. (2017)
	SpCas9	OsUbi	OsALS substitution	Agrobacterium Donor RNA fused with sgRNA			2.14	Bispyribac resistance	Butt et al. (2017)
	SpCas9	Maize Ubi	NRT1.1B	Bombardment Regenerate plants		By SpCas9	6.7 (14/233) Biallelic: 0.4 (1/223)	RFLP	Li et al. (2018a) and Li et al. (2018b)
	LbCas12a	Maize Ubi	OsALS substitution Only a left homology arm	Bombardment Regenerate plants		By LbCas12a	0.7 (1/152)	Bispyribac resistance	Li et al. (2018a) and Li et al. (2018b)
	LbCas12a	Maize Ubi	OsALS substitution	Bombardment Regenerate plants			RNA donor: 1.7 (1/58) DNA donor: 4.6 (4/87)	Bispyribac resistance	Li et al. (2019)
	LbCas12a	Maize Ubi	OsALS substitution	Bombardment Regenerate plants			1.8 (5/284) All biallelic	Bispyribac resistance	Li et al. (2019)
	SpCas9 fused with VirD2	OsUbi	OsALS OsCCD7 OsHDT	Agrobacterium			1.56–9.87	PCR	Li et al. (2020)
	SpCas9	Maize Ubi	19 loci NHEJ-mediated KI 5 loci TR-HDR	Bombardment Regenerate plants			NHEJ-mediated KI: 3.9–47.3 TR-HDR: 3.4–11.4%	PCR	Lu et al. (2020)
Maize	ZFN	Maize Ubi	IPK1 PAT-KI	Bombardment Regenerate plants			3.4–100	Basta resistance	Shukla et al. (2009)
	ZFN	Maize Ubi	PAT transgene ADD1-KI	Bombardment			3.1	Haloxyfop resistance	Ainley et al. (2013)
	SpCas9	Maize Ubi	ALS substitution LIG PAT-KI	Bombardment Regenerate plants			ALS: 2.4 LIG: 2.5–4.1	PCR	Svitashev et al. (2015)
	SpCas9		ALS2	Bombardment RNP Regenerate plants Transgene free			2–2.5	Bispyribac and PAT (Basta) resistance	Svitashev et al. (2016)
	SpCas9	Maize Ubi	ARGOS8 KI and replacement	Bombardment Regenerate plants			1–1.7	PCR	Shi et al. (2017)
	SpCas9	Hsp26	TS45	Agrobacterium		By SpCas9 Donor excision selection by Als	1.6	PCR	Barone et al. (2020)
Wheat	TALEN	Maize Ubi	MLO NHEJ-mediated GFP-KI	Protoplast electroporation		By TALEN	6.5	Flow cytometry	Wang et al. (2014)
	SpCas9	Maize Ubi	Ubi MLO EPSPS GFP- and BFP-KI	Protoplast electroporation	WDV		0.4–6.4	GFP and BFP fluorescent and flow cytometry	Gil-Humanes et al. (2017)
Potato	TALEN	CaMV 35S	ALS NptII-KI	Agrobacterium	Geminivirus		41.7 (5/12)	Kanamycin resistance	Butler et al. (2016)
	SpCas9						12.5 (1/8)		
Barley	TALEN	Maize Ubi	GFP transgene substitution	Bombardment			2–3	YFP fluorescent	Budhagatapalli et al. (2015)
Flax	SpCas9	MAS	EPES substitution	PEG-mediated transfection to protoplast Regenerate plants			0.08 (4/4,601) −0.15 (8/5,167)	Glyphosate resistance	Sauer et al. (2016)
Cassava	SpCas9	CaMV 35S	EPES KI	Agrobacterium			66.6 (4/6)	Glyphosate resistance	Hummel et al. (2018)

BS, bispyribac sodium.

## SSNs

Engineered SSNs, including ZFN, TALEN and CRISPR/Cas systems, generate DSBs at specific genomic target sites. ZFN and TALEN recognize their target loci via modified tandem repeat DNA-binding motifs, cleave DNA via the nonspecific DNA cleavage domain of the FokI endonuclease and require a pair of SSNs to generate a DSB at the target loci. The application of ZFN and TALEN to GT is complicated, and the efficiency of DSB generation is lower than that of CRISPR/Cas systems (Gaj et al. 2013).

Recently, the type II CRISPR/Cas9 from *Streptococcus pyogenes* (SpCas9) has been more widely used than ZFN and TALEN because of its higher DSB efficiency and its ease of use. In addition to the SpCas9 system, there have been many Cas9 orthologs identified in different bacteria. Reports show that a smaller Cas9 identified in *Staphylococcus aureus* (SaCas9) possesses higher DSB activity than SpCas9 (Wolter et al. 2018). Another widely used CRISPR/Cas system is the type V CRISPR/Cas12a (also known as Cpf1). Cas proteins interact with a single-guide RNA (sgRNA) to direct DNA cleavage. While type II Cas9 systems generate blunt end DSBs, type V Cas12a systems produce DSBs with 5′ overhang sticky ends. A protospacer adjacent motif (PAM) next to the target recognition site (protospacer) is required for DSB generation by CRISPR/Cas systems. The cleavage site of Cas9 systems is close to the PAM, but Cas12a systems cleave at a site distal to the PAM sequence. Based on this difference in cleavage sites, researchers have speculated that mutations generated by error-prone NHEJ interfere with further cleavage by Cas9 systems but interfere less with Cas12a cleavage (Wolter and Puchta 2019).

Several modifications to Cas9 and Cas12a systems have been generated to improve their usability and efficiency. Endo et al. (2019) reported that, in rice, modified SpCas9 (SpCas9-NGv1) recognizes the NG PAM sequence, while wild-type (WT) SpCas9 recognizes the NGG PAM sequence (Endo et al. 2019). However, this SpCas9-NGv1 was not used for GT because its DSB efficiency, which is important for successful GT, was lower than that of the WT SpCas9. Another example is high temperature-tolerant LbCas12a (ttLbCas12a). Currently, two Cas12a systems, *Labchnospiraceae bacterium ND2006* Cas12a (LbCas12a) and *Francisella novicida* Cas12a (FnCas12a), are widely used in higher plants (Wang et al. 2018). However, the activity of these systems is relatively low because the temperatures required for their activity are too high for plant growth (Malzahn et al. 2019). Based on attempts by Kleinstiver et al. (2019) to increase the Cas12a nuclease activity in human cells, Schindele and Puchta (2020) generated a highly efficient temperature-tolerant mutant of LbCas12a (ttLbCas12a) with a single amino aside substitution, i.e. D156R; this mutant had higher mutation efficiency than the wild-type LbCas12a in Arabidopsis at growth temperatures of both 22 and 28°C (Kleinstiver et al. 2019, Schindele and Puchta 2020).

From 2005 to 2016, ZFN-mediated GT was reported in Arabidopsis (de Pater et al. 2013, Qi et al. 2013, Weinthal et al. 2013, Baltes et al. 2014), tobacco (Wright et al. 2005, Cai et al. 2009, Townsend et al. 2009, Baltes et al. 2014, Schneider et al. 2016) and maize (Shukla et al. 2009, Ainley et al. 2013). From 2013 to 2017, TALEN-mediated GT was reported in Arabidopsis (Sauer et al. 2016), tobacco (Zhang et al. 2013), tomato (Čermák et al. 2015), rice (T. Li et al. 2016), wheat (Wang et al. 2014), potato (Butler et al. 2016) and barley (Budhagatapalli et al. 2015). Since 2013, Cas9 has been the most widely used SSN system for GT, because of its higher DSB efficiency. Cas12a systems have also been widely used for GT in Arabidopsis (Wolter and Puchta 2019), tomato (Vu et al. 2020) and rice (Begemann et al. 2017, Li et al. 2018, Li et al. 2019, Li et al. 2020c). Interestingly, comparisons of orthologous systems revealed that FnCas12a had higher GT efficiency than LbCas12a in rice (Begemann et al. 2017) and that SaCas9 had higher GT efficiency than SpCas9 in Arabidopsis (Wolter et al. 2018). Researchers recently reported that ttLbCas12a, in addition to having a higher mutation efficiency, has a higher GT efficiency than WT LbCas12a in Arabidopsis and tobacco (Merker et al. 2020b, Huang et al. 2021). GT by SSNs in various plant species is summarized in Table 1. In addition to the SSNs described above, meganuclease-mediated GT in an integrated T-DNA locus was reported in cotton (D'Halluin et al. 2013).

## A Straightforward ‘All-in-One’ Strategy

Efficient GT in plants requires both an SSN for generating a DSB at a specific target site and a repair donor template. Most published examples involve the use of an ‘all-in-one’ T-DNA construct that contains both the SSN system and the donor template for *Agrobacterium* transformation (Table 1). In the case of biolistic transformation, the SSN system and donor template are simultaneously delivered into embryonic tissues, calli or protoplasts by bombardment or electroporation (Table 1). An all-in-one strategy in Arabidopsis, e.g. involves a T-DNA construct that contains SpCas9 driven by the strong constitutive CaMV 35S promoter, an sgRNA driven by the AtU6-26 promoter and a donor DNA sequence. Although some publications report successful GT in higher plants using this straightforward all-in-one strategy (Table 1), additional improvements are required to increase its efficiency in higher plants.

## Tissue-Specific Promoters

Except in Arabidopsis, strong constitutive promoters (such as CaMV 35S, ubiquitin and EFlA2) were mainly used to drive SSNs for GT establishment in plants (Table 1). To obtain heritable GT in plants, the HR must be established in germline cells or at the very beginning of transformation. Thus, these strong constitutive promoters must be highly efficient in establishing GT at an early time during the transformation in crop plants. Unfortunately, the activity of the most frequently used CaMV 35S promoter is weaker in meristem and germline cells than in other somatic cells in Arabidopsis (Ge et al. 2008). Several attempts have been made to use germline-specific promoters to achieve heritable GT in Arabidopsis. Heritable GT plants were successfully obtained using a chimeric egg cell-specific Egg Cell 1.1 (EC1.1) promoter with the EC1.2 enhancer to drive SaCas9, LbCas12a and ttLbCas12a in the endogenous *ALS* locus in Arabidopsis (Wolter et al. 2018, Wolter and Puchta 2019, Merker et al. 2020a). Similarly, the use of the egg cell- and early embryo-specific DD45 (also known as EC1.2) promoter to drive SpCas9-facilitated GT establishment in the endogenous *ROS1 and DME* loci in Arabidopsis (Miki et al. 2018, Peng et al. 2020). However, use of the EC1.1 promoter with the EC1.2 enhancer to drive the SpCas9 system failed to establish GT in at least one study (Peng et al. 2020).

Other germline- or meristem-specific promoters have also been investigated in Arabidopsis. Driving Cas9 with a pollen-specific promoter from tomato (Lat52) or with meristem-specific promoters CLAVATA3 (CLV3), YAO and CDC45 from Arabidopsis failed to establish efficient heritable GT in Arabidopsis (Miki et al. 2018, Wolter et al. 2018). However, Wolter et al. (2018) reported that YAO promoter-driven SaCas9 produced heritable GT, albeit with lower efficiency than the *Petroselinum crispum* ubiquitin 4-2 (PcUbi4-2) promoter (Table 1). These results suggest that efficient heritable GT should be established in the egg cell and/or early embryo via *Agrobacterium* transformation in Arabidopsis because egg cells are the primary target of *Agrobacterium* infection when the floral dip transformation method is used (Bent 2000).

Alternatively, a heat shock protein 26 (Hsp26) promoter from maize was recently used to establish heritable GT in maize (Barone et al. 2020). SpCas9 expression was induced 4 or 11 d after *Agrobacterium* infection by heat shock. Barron et al. obtained NptII knock-in lines in the endogenous TS45 locus with both induction timings, suggesting that DSB-mediated GT was established via activation of SpCas9 transcription by heat shock. Heat shock might also increase the targeted mutagenesis activity of SpCas9 (LeBlanc et al. 2018). Taken together, the application of a heat shock-inducible promoter for driving Cas9 expression can be a preferred option for the establishment of heritable GT in higher plants.

The most commonly used promoter to establish GT in crop plants has been the CaMV 35S promoter (Table 1). We hypothesize that effective generation of DSBs at the time of *Agrobacterium* infection may be important for establishing heritable GT, and thus, the promoters most robustly express SSNs in the precise tissues where *Agrobacterium* infection or biolistic transformation occurs are important.

## Transcriptional and Translational Enhancers

Transcriptional and translational enhancers have been reported to increase Cas9 expression and the efficiency of mutagenesis in plants. The Arabidopsis transcriptional enhancer AtUBQ10 (a 5′ UTR sequence with the 1st intron from UBQ10), e.g. improved the mutation efficiency of several CRISPR/Cas9 target regions in barley (Gasparis et al. 2018); germline-specific SpCas9 expression from the SPL promoter in Arabidopsis was increased by a 2× transcriptional enhancer element from the CaMV 35S promoter (Mao et al. 2016); and dMac3 (a rice translational enhancer) increased mutagenesis frequency by multiple sgRNAs in potato (Kusano et al. 2018). The EC1.2 transcriptional enhancer also enhanced the efficiency of SpCas9-mediated mutagenesis and SaCas9-mediated GT in Arabidopsis (Wang et al. 2015, Wolter et al. 2018). Furthermore, the EC1.1 promoter with the EC1.2 enhancer system was also applied to LbCas12a and ttLbCas12a, achieving efficient GT in Arabidopsis (Wolter and Puchta 2019, Merker et al. 2020a). LbCas12a-mediated GT was reported in tomato, although the authors did not compare the GT frequency with or without the AtUBQ10 transcriptional enhancer (Vu et al. 2020). An omega translational enhancer from tobacco mosaic virus improved SpCas9-mediated GT by least 3-fold in Arabidopsis (Peng et al. 2020). As we speculated earlier, DSB efficiency by SSNs is likely one of the most important factors for establishing heritable GT. As a consequence, transcriptional and translational enhancers may increase GT frequency. Together, the results described above indicate that simple enhancement of SSN expression can increase GT frequency in higher plants. Further work is required to investigate the use of other enhancer combinations with the goal of establishing GT in crop plants.

## Sequential Transformation

The sequential transformation method involves the transformation of parental plants already expressing Cas9 (or other CRISPR/Cas systems) with a construct containing one of the following: (i) a GT donor sequence, (ii) an sgRNA targeting a genomic locus of interest and (iii) a selectable marker. This method could theoretically be used to establish GT in crop plants. Sequential transformation has been reported in three publications to date, two of which used the ZmUbi promoter to drive SpCas9 in rice (Endo et al. 2016, Wang et al. 2017), and one of which used the DD45 promoter to drive SpCas9 in Arabidopsis (Miki et al. 2018). All three publications reported that heritable GT establishment efficiency was greatly improved by the sequential transformation method. Plants showing the highest DSB efficiency, reflected by SpCas9 activity, are selected for use as the parental line for sequential transformation. By contrast, SpCas9 or other Cas protein activity is presumably variable among independent transgenic lines when the all-in-one method is used. This is the main reason why the sequential transformation increases GT frequency. Although sequential transformation is useful for the generation of heritable GT in crop plants, it requires that parental lines are genetically modified and already expressing Cas, which limits its applicability in diverse genetic backgrounds.

## Genetic Factors

Several cellular components in the host plants are required for efficient HDR. Researchers have also reported that HDR frequency is increased in many organisms when the NHEJ pathway is blocked. KU70 and LIG4 are highly conserved among kingdoms and are important components in the NHEJ pathway. GT efficiency was enhanced in an *lig4* mutant, and a *ku70* mutant showed the highest ZFN-mediated GT frequency in Arabidopsis protoplasts (Qi et al. 2013). Similarly, SpCas9-mediated GT frequency was increased in a rice *lig4* mutant background (Endo et al. 2016). Another factor, SMC6B, is involved in sister-chromatid-based HR; mutation of SMC6B in Arabidopsis protoplasts increased GT efficiency compared to the WT (Qi et al. 2013). An alternative approach is to overexpress proteins involved in the HDR pathway. RAD proteins are involved in both DNA DSB repair and HDR; overexpression of AtRad52-1A increased meganuclease I-SceI-mediated intrachromosomal HR in Arabidopsis nuclei (Samach et al. 2018). On the other hand, LbCas12a-mediated GT efficiency was not changed when SlRAD51 or SlRAD54 was over-expressed in tomato (Vu et al. 2020). Intriguingly, mutants of HR suppressor genes, such as *rtel1*, *rmi2* and *fancm1*, did not show improvement in LbCas12a-mediated GT efficiency in Arabidopsis (Wolter and Puchta 2019). While enhancement of GT efficiency was observed in NHEJ pathway mutants, these mutations frequently resulted in the accumulation of spontaneous mutations in the genome. In addition, overexpression of heterologous recombinases frequently caused morphological defects of plants (Barakate et al. 2020). Thus, although these manipulations have potential for application to crop plants, some refinements will be required to avoid unwanted consequences.

## Donor Template

As described above, some of the most important factors for GT establishment are efficiency and timing of DSBs. Additional critical factors are the delivery and copy number of the donor template. It is generally accepted that increasing the copy number of the donor template will enhance GT frequency. An excess of double-stranded (ds) or single-stranded (ss) DNA or RNA donor template can be delivered via biolistic transformation, but the delivery of too many copies of templates results in unwanted, random integration into the genome and reduced GT efficiency; using RNA as the donor template further reduced GT efficiency (Li et al. 2019). As an alternative, virus replicons have been used to increase the copy number of the donor template for *Agrobacterium* transformation in Arabidopsis (Baltes et al. 2014), tobacco (Baltes et al. 2014), tomato (Čermák et al. 2015, Dahan-Meir et al. 2018, Vu et al. 2020), rice (Wang et al. 2017), wheat (Gil-Humanes et al. 2017) and potato (Butler et al. 2016) (Table 1). The geminivirus replication system requires three elements: (i) the *trans*-acting replication protein Rep/RepA, (ii) the *cis*-acting large intergenic region and (iii) the short intergenic region sequences. All three components must be delivered into the plant cell for the amplification and accumulation of circular geminivirus replicons (Baltes et al. 2014). The *Agrobacterium* T-DNA must therefore contain these three elements from geminivirus plus the SSN system expression cassette(s) and the donor sequence. While the detailed mechanism by which the virus replicon system can facilitate GT is unclear, we speculate that at least two factors may be involved: (i) the system increases the copy number of the donor template and (ii) Rep/RepA facilitates DNA replication and GT itself (Huang and Puchta 2019). Although the virus replicon system may prove to be useful for establishing efficient GT in crop plants, two publications reported that the virus replicon system failed to increase GT efficiency in Arabidopsis (de Pater et al. 2018, Hahn et al. 2018). This suggests that the virus replicon system is not guaranteed to increase GT efficiency in plants and that more research is required to improve its use.

Another approach to enhance GT is to excise the donor template from randomly integrated T-DNA. The SSNs are designed not only to generate a DSB (or DSBs) at the target locus, but also at both ends of the donor template, and excision of the donor template is expected to enhance the physical interaction between the DSB target locus and a donor template DNA fragment. This donor template excision system has helped to achieve GT in Arabidopsis (Fauser et al. 2012, Schiml et al. 2014, Zhao et al. 2016, Hahn et al. 2018, Wolter et al. 2018, Wolter and Puchta 2019, Merker et al. 2020a), rice ( Li et al. 2018a, Li et al. 2018b), tobacco (Huang et al. 2021) and maize (Barone et al. 2020). However, excised genomic DNA fragments are expected to be easily degraded by endogenous DNase activities. One publication reported failure to improve GT in Arabidopsis if the donor template was excised (Peng et al. 2020). Alternatively, the donor excision events were taken advantage of selection for possible GT and GT maize was successfully obtained (Barone et al. 2020). Further work is needed to evaluate the strategy of excision of the donor template from T-DNA to increase GT efficiency.

In addition to DNA, RNA can be a donor template for HDR-mediated GT (Li et al. 2019). A biolistic approach is most commonly used to introduce the RNA donor template into plant cells, although Butt et al. (2017) used a strategy in rice in which the GT donor template RNA was fused with sgRNA. This guide RNA molecule is referred to as a chimeric single-guide RNA (cgRNA) and can be transcribed and accumulated at higher copy number than a DNA sequence. Furthermore, the cgRNA is delivered near the DSB site by SpCas9. Although it requires additional development, the cgRNA system appears to be an interesting option to obtain GT in crop plants.

**Fig. 2:**
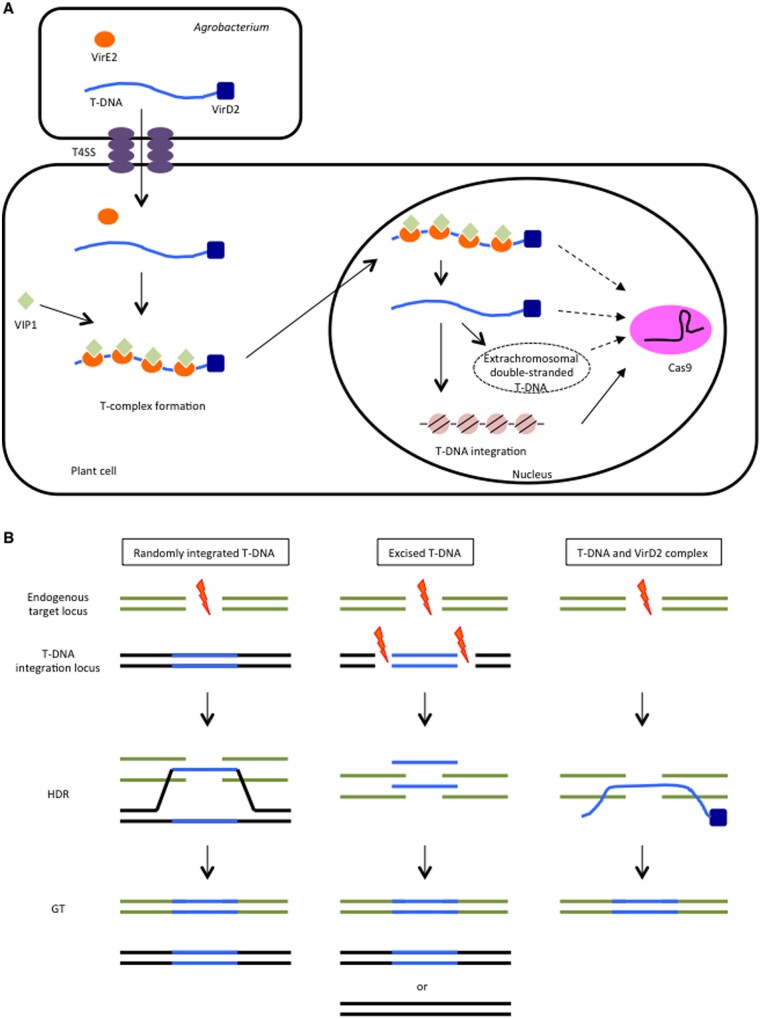
Possible working models of *Agrobacterium* transformation-mediated gene-targeting in plants. (A) T-DNA integration into the host plant genome. The single-stranded T-DNA region of the Ti plasmid forms a complex with VirD2, which along with other Vir proteins is transported to plant cells by the T4SS. VIP1, an Arabidopsis bZIP protein, together with *Agrobacterium* VirE2, forms a T-complex that is imported into the nucleus. In the nucleus, VirE2 and VIP are detached from the single-stranded T-DNA and VirD2 complex, and the T-DNA is integrated into the host plant genome. The SSNs, Cas9 and sgRNA are transcribed from the integrated T-DNA locus (or loci), although in some rare instances they might also be transcribed from nonintegrated T-DNA, such as extrachromosomal ds T-DNA (Gelvin 2017, Nishizawa-Yokoi and Toki 2021). (B) HDR-mediated GT pathways in plants. DSBs generated by SSNs are repaired via HDR. There are three possible repair templates: (i) randomly integrated T-DNA in a different locus, (ii) excised donor fragment from the integrated T-DNA locus and (iii) single-stranded T-DNA with VirD2.

Although large amounts of donor template DNA and RNA fragments can be introduced into plant cells via biolistic transformation, the most efficient donor template for *Agrobacterium*-mediated plant GT remains unclear. We speculate that it might be the complex formed by the free ssT-DNA and *Agrobacterium* VirD2 protein. *Agrobacterium* transfers excised ssT-DNA with the VirD2 complex and VirE2 protein through the T4SS system to the infected plant cell. The host plant factor VIP1 and *Agrobacterium* VirE2 associate with the ssT-DNA and VirD2 complex to form the mature T-complex in the cytoplasm; this complex is transported into the nucleus, where VIP1 and VirE2 release the ssT-DNA and VirD2 complex, and T-DNA is integrated into the host plant genome (Fig. 2A). The ssT-DNA and VirD2 complex accumulates in the nucleus, and we hypothesize that it may be an efficient GT donor template (Fig. 2B). Our hypothesis was supported by Ali et al. (2020), who found that the efficiency of GT was much greater with the SpCas9–VirD2 fusion protein than with the WT SpCas9 in rice. VirD2 has been sometimes used as a nuclear localization signal. The SSNs–VirD2 fusion protein may be very useful for GT in crop plants. We recognize that the already integrated T-DNA, excised T-DNA fragment and free ssT-DNA with VirD2 complex may all be used as HDR templates for GT in plant cells (Fig. 2B).

## Alternative Approaches

In this review, we have focused on SSN-facilitated and HDR-based GT in higher plants. SSN-facilitated but HDR-independent precise genome modification methods, however, have also been developed; these are briefly introduced in the section below.

First, NHEJ-mediated knock-ins have been reported in rice and wheat (Fig. 1). J. Li et al. (2016) replaced the second exon of the endogenous rice gene 5-enolpyruvylshikimate-3-phosphate synthase (*OsEPSPS*) using two sgRNAs to target the first and second introns. In addition, a TALEN-mediated, epitope-tagged knock-in was reported at the mildew-resistant locus (*TaMLO*) in bread wheat protoplasts (Wang et al. 2014). Researchers recently reported that phosphorothioate-linkage and 5′-phosphorylation modifications in donor DNA increase SpCas9-mediated knock-in efficiency in rice (Lu et al. 2020). The phosphorothioate-linkage modification prevents degradation of DNA fragments in cells. The authors performed knock-ins with enhancer sequences (26–2,049 bp in length) into 14 genetic loci with an average efficiency of 14%. They also achieved precise knock-in and replacement via NHEJ followed by tandem repeat-HDR (TR-HDR) in 4 loci. The NHEJ-mediated knock-in system, however, frequently generates short in-del mutations at both junctions of the insertion sequence, and the knock-in donor fragment can be inserted in both orientations (Fig. 1) (Wang et al. 2014, Li et al. 2016, Lu et al. 2020). Thus, intron regions or the 5′ UTR sequence, which may not affect the functions of the coding sequence, are preferred targets of the SSNs to establish NHEJ-mediated knock-ins (Li et al. 2016, Lu et al. 2020).

Another approach involves prime editors (PEs) and was originally developed in human cells to generate precise genome editing without the requirement for DSBs and donor DNA templates (Anzalone et al. 2019). After a reverse transcriptase (RT)–SpCas9 nickase (nCas9) fusion protein binds to a prime editing guide RNA and generates site-specific nicking, RT introduces the desired mutations. PE-mediated base conversions and small insertions were also reported in rice, wheat and tomato (Hua et al. 2020, Li et al. 2020a, Lin et al. 2020, Tang et al. 2020, Xu et al. 2020a, Xu et al. 2020b, Lu et al. 2021). Thus, PE is potentially a powerful tool for precise base conversions, although it seems to have limited applications for introducing precise insertions, especially insertions of long sequences (Hassan et al. 2020).

## Epigenetic Modifications

Because DNA methylation and histone modifications contribute to the regulation of gene activity and stability, determining whether any epigenetic modifications are altered by GT events at the target locus is important; unfortunately, GT-associated alterations in epigenetic status have rarely been considered. A reduction in DNA methylation was observed with ZFN-mediated GT of the endogenous *PPOX* locus in one Arabidopsis line (Lieberman-Lazarovich et al. 2013). In the same study, however, the authors found no alteration in DNA methylation at the *PPOX* locus in two other lines or in GT lines of an mRFP knock-in at the *CRUCIFERIN3* locus. In another report, CRISPR/Cas9-mediated GT did not affect the cytosine methylation status of the endogenous target locus *ROS1* (Miki et al. 2018). New DNA methylation was not established *de novo* at the knock-in sequence in Arabidopsis (Peng et al. 2020). Taken together, these studies suggest that epigenetic status, particularly DNA methylation, mostly is not altered by GT events in Arabidopsis.

## Perspectives

CRISPR/Cas9 and other SSNs have been widely used in plant biotechnology to improve crop traits (Hua et al. 2019, Kaul et al. 2020, Zhu et al. 2020). Such genome-edited (GE) crops are mutagenized at a specific target locus (or loci) by SSNs, and the transgenes containing the SSN expression cassette can be removed by back-crossing of the generated GE plants. Transgenes, by contrast, must be retained by genetically modified (GMO) plants to retain the improved traits. A key question is whether GE crops should follow GMO regulation policies; the answer will determine whether GE crops will be subject to product-based or process-based GMO regulation policies. The US Department of Agriculture ruled that GE crops (mushroom and corn) were not subject to traditional GMO regulation (Waltz 2016). By contrast, GE plants have been considered as equivalent to GMOs by the Court of Justice of the European Union (ECJ) and are subject to process-based GMO regulation (Callaway 2018). A possible solution to this conflict is to use ribonucleoproteins (RNPs), which are in vitro assembled gRNA and CRISPR/Cas protein complexes. Svitashev et al. (2016) obtained GT maize plants using biolistic delivery of SpCas9-sgRNA RNP and an ss DNA oligo as a repair template into maize embryonic cells. Moreover, transgene integration-free GT tomato (Danilo et al. 2019) and tobacco (Huang et al. 2021) were achieved via *Agrobacterium*-mediated transformation. These results indicate that transgene integration-free GT crops can be generated, although an efficient screening method is required to identify potential GT events.

Another concern is that GT crops harbor a donor template-derived sequence (either a sequence replacement or a knock-in). SSN plants, i.e. GE plants generated by site-directed nucleases (SDNs), can be categorized into three groups (EFSA Panel on Genetically Modified Organisms 2012): in SDN-1, the DSB is repaired without involvement of a donor template; in SDN-2 and SDN 3, the repaired DSB generates short sequence replacements/insertions with <20 nucleotides (for SDN-2) or with >20 nucleotides (for SDN-3) (Hilscher et al. 2017, Gao 2018, Kaul et al. 2020). However, the plant’s own sequences can also be replaced or inserted at the target genomic site using GT; thus, a broader discussion of the SDN-2 and SDN-3 categories is required.

We expect that highly efficient and simple GT technologies will be developed and applied to crop breeding in the near future. The GT technique can improve gene function in ways that differ from simple mutation-based SSN-mediated breeding, although for most proteins the fundamental knowledge is still lacking regarding how to improve protein function. More research is required for the widespread application of GT techniques for the improvement of crops.
